# Open Reduction and Internal Fixation With Proximal Humerus Locking Plate, Screws, and Fibular Strut Graft: An Alternative to Shoulder Arthroplasty and Solution to Medial Instability and Varus Collapse in Comminuted Proximal Humerus Fractures

**DOI:** 10.7759/cureus.53164

**Published:** 2024-01-29

**Authors:** Horea Pop, Andrei Morcovescu, Maria M Mihai, Andrei S Constantinescu, Florin Bica

**Affiliations:** 1 Orthopedics and Traumatology, Bagdasar-Arseni Emergency Clinical Hospital, Bucharest, ROU

**Keywords:** fibular strut graft, proximal humerus internal locking system, varus collapse, medial calcar, proximal humerus fracture, philos plating

## Abstract

Fractures of the proximal humerus are a frequent ailment in the senior population. One concern with such a case that presented in our emergency room was the risk of varus collapse following open reduction and internal fixation (ORIF) in a patient with marked calcar comminution. The surgical method used in this case of a NEER four-part fracture with medial column instability proved effective in terms of good surgery outcome, patient satisfaction, good range of motion, and low or no pain at post-surgical follow-ups. ORIF with a locking plate and fibular strut graft proved to be a viable alternative to solo plating or shoulder arthroplasty. We conducted upper extremity patient-reported functional outcome scales (ASES, CONSTANT, and Neer’s “limited goals”) with this particular patient. At the last visit, two years after the first surgical intervention, the patient had a normal range of motion, was pain-free, and was generally satisfied with the results of the surgery. We concluded that ORIF of the proximal humerus with locking plate and fibular strut graft was, in our case, a good solution for preventing varus malalignment in a patient with severe comminution of medial calcar.

## Introduction

Fracture of the proximal humerus is the third most common fracture in the elderly population, not including vertebral pathology, and the most frequent concerning the shoulder girdle [1]. Its prevalence is in the elderly population (80-89 years old), between 52 and 56 times more frequent than in the young population (20-29 years old). Even though the incidence has been shown to increase steadily over the years, the elderly female population remains the most frequently affected [1]. A proximal humerus fracture can prove highly debilitating, with pain scores in the upper extreme, almost no range of motion in the shoulder, and a great impact on the ability to carry out normal daily activities.

When trying to classify the proximal humerus fractures using imaging findings, the NEER classification states that a fracture is deemed as displaced when the fragments are shifted by more than 1 cm or have an angulation of at least 45° [1]. NEER four-part fractures exhibit a shift or angulation in all the segments: greater and lesser tuberosity, head of the humerus, and shaft of the humerus [1].

Displaced fractures of the proximal humerus continue to be challenging due to the difficulty of achieving stable fixation with maintainable intraoperative reduction [2]. Various operative choices, such as plates with conventional screws, locking plates, or hemiarthroplasty, have each shown their respective drawbacks. Plates with conventional screws have shown hardware loosening or varus collapse, locking plates have shown cutout or screw penetration [1,3], and some studies have reported lower Constant scores in hemiarthroplasty compared to open reduction and internal fixation (ORIF), while other studies have shown similar results in scores [1]. Regarding reverse total shoulder arthroplasty, the preferred patient with comminuted proximal humerus fracture is one with a history of rotator cuff deficiency or omarthrosis. Even though humeral head osteonecrosis can be a common adverse outcome in most ORIFs, it was shown that partial necrosis is admissible and similar in patient outcome and satisfaction to that of hemiarthroplasty [1]. This being said, it is considered that every effort must be taken to attempt an acceptable ORIF fixation, especially in the young population, before arthroplasty is considered [1].

In cases of medial column comminution, it was shown that acceptable mechanical support may be offered by the use of a fibular autograft in combination with a locking plate [3]. The fibular graft is also often very useful in aiding with reduction. This construct should be able to prevent varus malalignment [1].

The purpose of our case study was to show that the use of a fibular autograft placed in the humeral canal, in combination with a locking plate, proved to be a solution for preventing medial instability and varus collapse in our patient, provided good surgical outcomes, patient satisfaction, and prevented or postponed the need of arthroplasty.

## Case presentation

Materials and methods

Our study centers around the post-operative evolution of a 63-year-old female patient with NEER four-part right proximal humerus fracture, treated with ORIF with locking plate and fibular strut autograft. The patient was informed about this study, consent was obtained, and approval from the institutional review board was granted.

**Figure 1:**
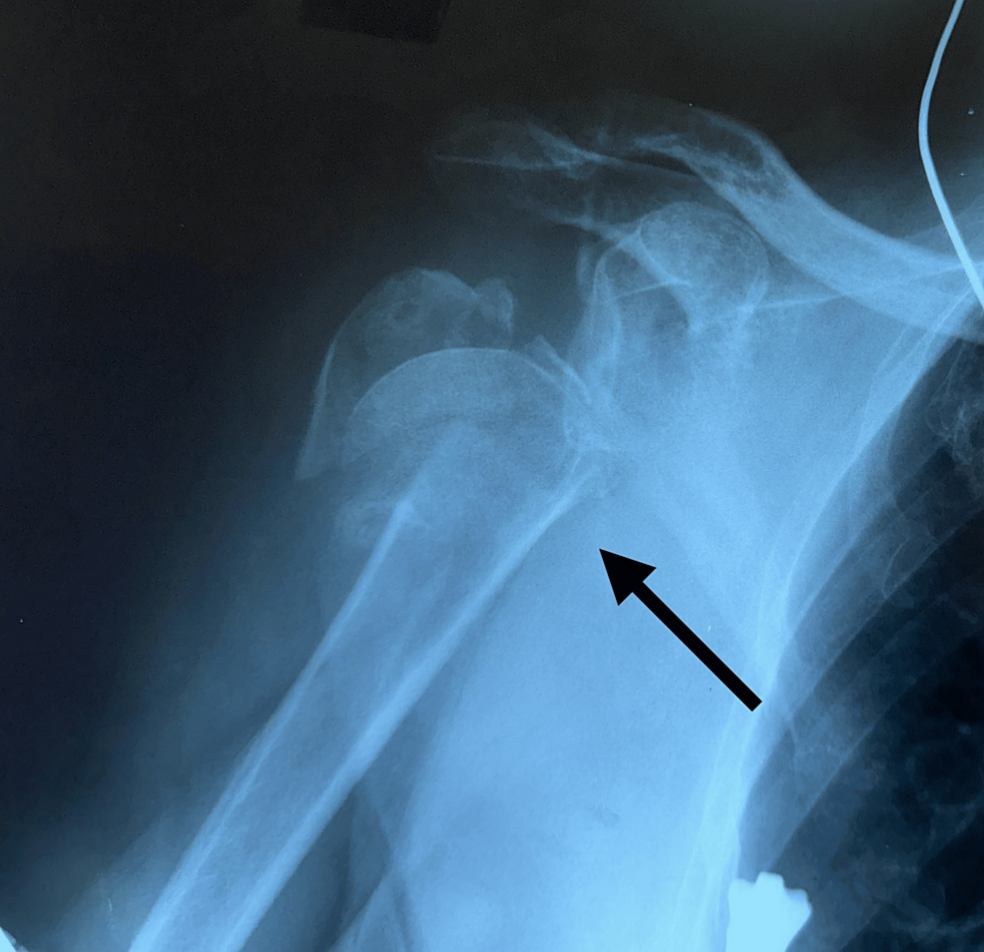
AP X-ray of the right shoulder with visible proximal humerus fracture The arrow shows a fracture site with NEER four-type fracture: displacement and angulation of greater and lesser tuberosity and head of the humerus in relation to the humerus shaft.

The patient presented at the emergency department of our hospital with pain and loss of function in her right shoulder after sustaining an accidental fall two days prior. Physical examination showed right shoulder and arm ecchymosis, swelling, and pain when trying to passively or actively mobilize the shoulder. The patient had extreme pain, reporting a pain level of 8 on a 1-10 pain scale. There was no neurovascular damage noted. Clinically and radiologically, it was determined that the patient had a comminuted fracture of the proximal right humerus (Figure 1). Anteroposterior (AP) X-ray of the right shoulder shows NEER four-part fracture (displacement and angulation of greater and lesser tuberosity and head of humerus in relation to humerus shaft).

The patient had a history of hypertension, dyslipidemia, and ischemic heart disease and was under treatment with candesartan, amlodipine, metoprolol, atorvastatin, nicergoline, and clopidogrel. The surgery was postponed seven days due to the need to transition to low molecular weight heparin. The patient was treated conservatively with a Desault bandage and was admitted into the hospital five days after the first presentation in the emergency unit and a total of seven days after the traumatism. The surgical intervention was carried out three days after admission.

**Figure 2:**
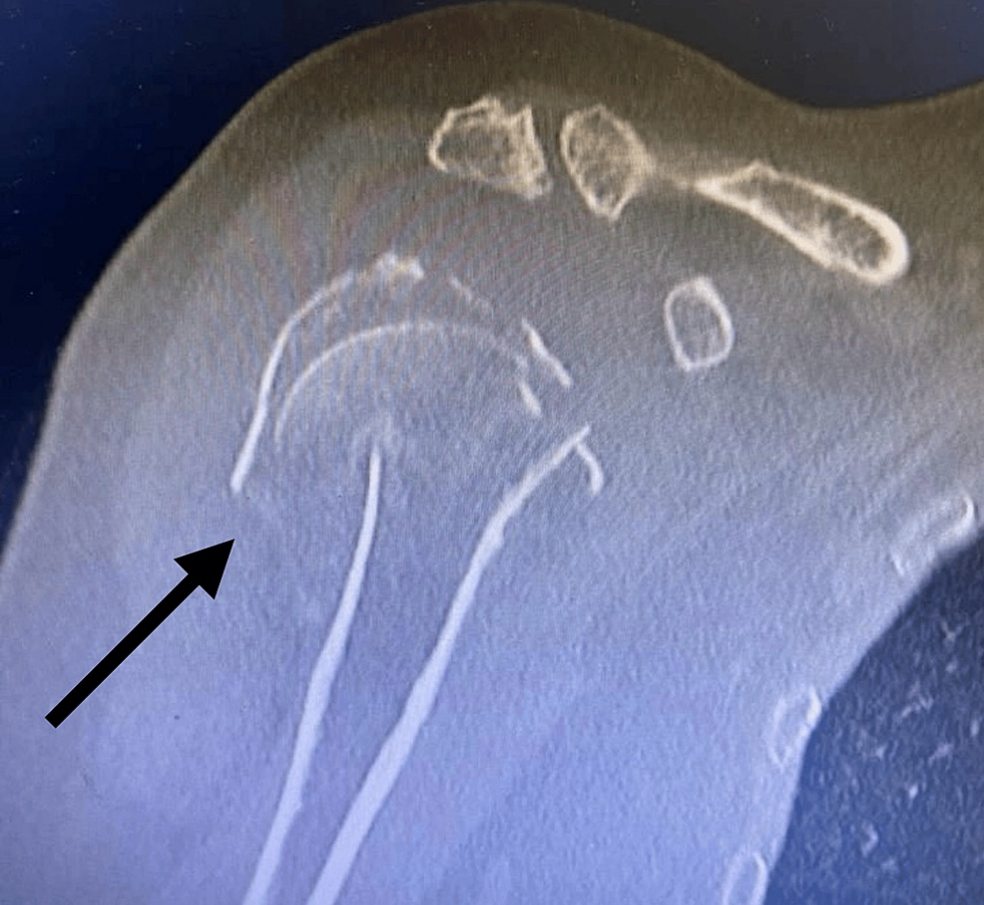
CT scan of the right shoulder The arrow shows a fracture site with NEER four-type fracture: displacement and angulation of greater and lesser tuberosity and head of the humerus in relation to the humerus shaft.

The patient was evaluated preoperatively with a plain AP X-ray and computed tomography (CT) scan of the afflicted shoulder (Figure 2).

The patient returned for follow-ups at two weeks, four weeks, three months, six months, 12 months, 18 months, and two years after surgery to assess pain level, range of motion, and construct stability by clinical and radiological evaluations. We assessed the patient radiologically for construct integrity and bone mass structure and with various functional outcome scales: ASES, CONSTANT, and Neer’s “limited goals” at six weeks, six months, one and a half years, and two years after surgery.

Surgical technique

**Figure 3:**
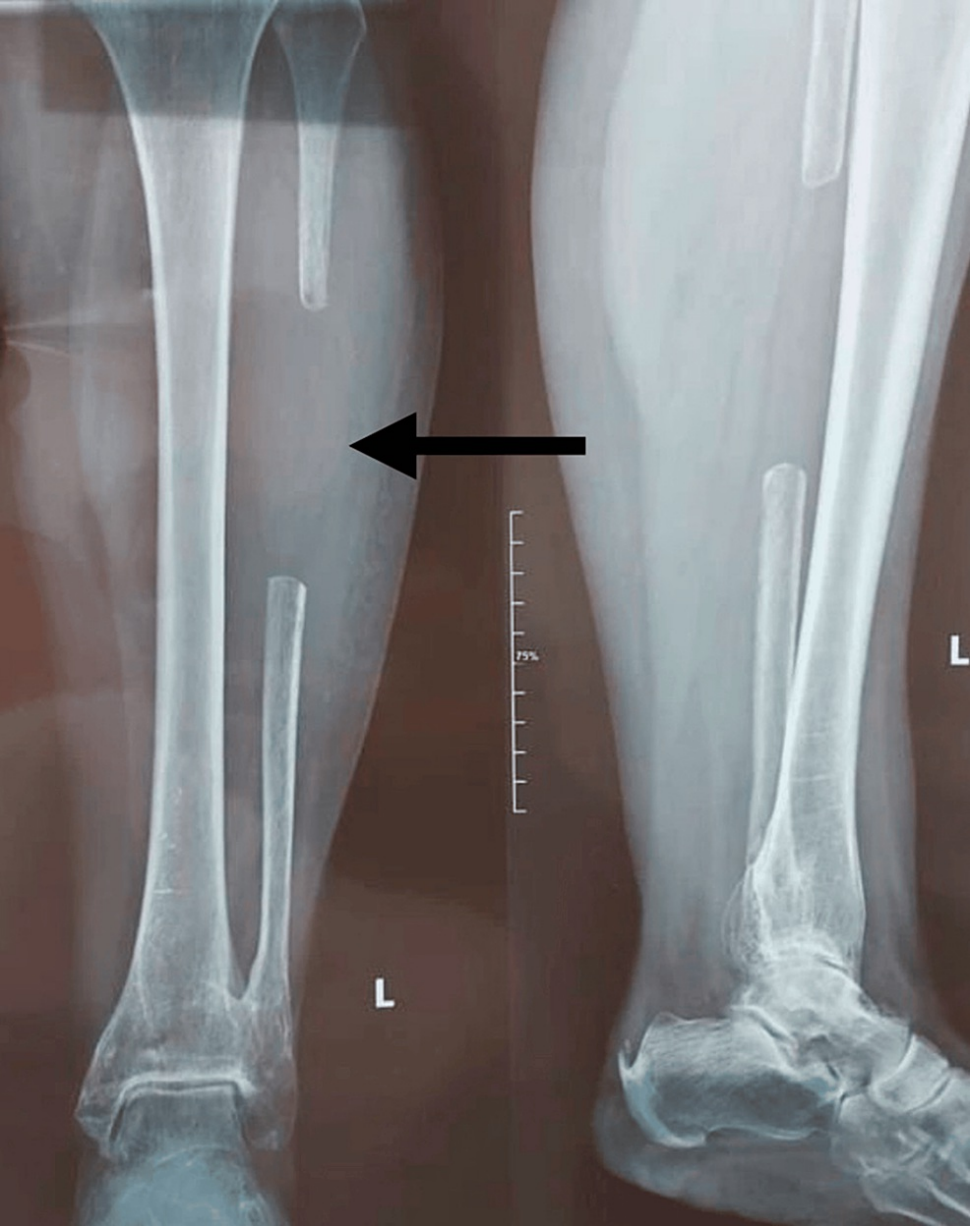
Postoperative AP and lateral X-ray of patient’s contralateral (left) leg showing fibular autograft donor site The arrow shows the donor site located in the shaft of the left fibula.

For our procedure, the patient received general anesthesia after being placed in a supine position on a radiolucent operating table. After the proper antiseptic techniques and steps and sterile draping were done, a deltopectoral approach was used for the right shoulder, careful plane-by-plane dissection was carried out, long head biceps tenotomy was performed, and exposure of fracture fragments was made. At the same time, the fibula autograft was being extracted by colleagues from the plastic surgery department (Figure 3). The fibular shaft autograft was harvested from the contralateral leg via a lateral approach, avoiding the proximal and distal 5-10 cm of the fibula. Approximately 8 cm of fibula shaft was harvested.

Non-absorbable size two sutures were placed in the rotator cuff tendons to aid traction and reduction of fracture. These were later exchanged with high-strength sutures. The humeral medullary canal was prepared to accommodate the graft. On the instrumentation table, the fibular graft was stripped of the periosteum and adjusted to optimal length, which in our case was 6.5 cm. This length might differ in other patients; the surgeon must adjust the graft length so that it fits in the humerus canal while also permitting proper fracture reduction. The fibular autograft was then placed in the humeral shaft canal and retrogradely inserted into the subchondral bone of the humeral head. The fragments were temporarily held in place with K-wires. A locking plate was then placed on the anterolateral aspect of the proximal humerus, lateral to the bicipital groove. First, a non-locking screw was placed to aid in plate placement and adjustment, and then, locking screws were inserted. The first screw was later exchanged for a locking screw. The rotator cuff insertions were reattached to the humeral head and secured to the plate with high-strength sutures, long head biceps tenodesis was performed, and the operative wound was sutured. Antibiotic therapy consisted of one dose of 2 g i.v. Ceftriaxone at the admission of anesthesia and then two more doses of ceftriaxone: one dose of 1 g i.v. at 12 hours postoperatively and the second dose of 1 g i.v. at 24 hours postoperatively. Concerning postoperative care, the patient was given the following recommendations: passive pendular movements for the first three weeks and sling immobilization between the passive pendular movement sessions for the first two weeks. At the three-week mark, the patient started assisted abduction and forward flexion. At six weeks postoperatively, the patient started slight resistance exercises, and at the nine-week landmark, she started isotonic, eccentric, and concentric exercises. Overall, the patient avoided external rotation and resistance exercises for six weeks postoperatively. The patient was also prescribed low molecular weight heparin for three weeks postoperatively.

Results

**Figure 4:**
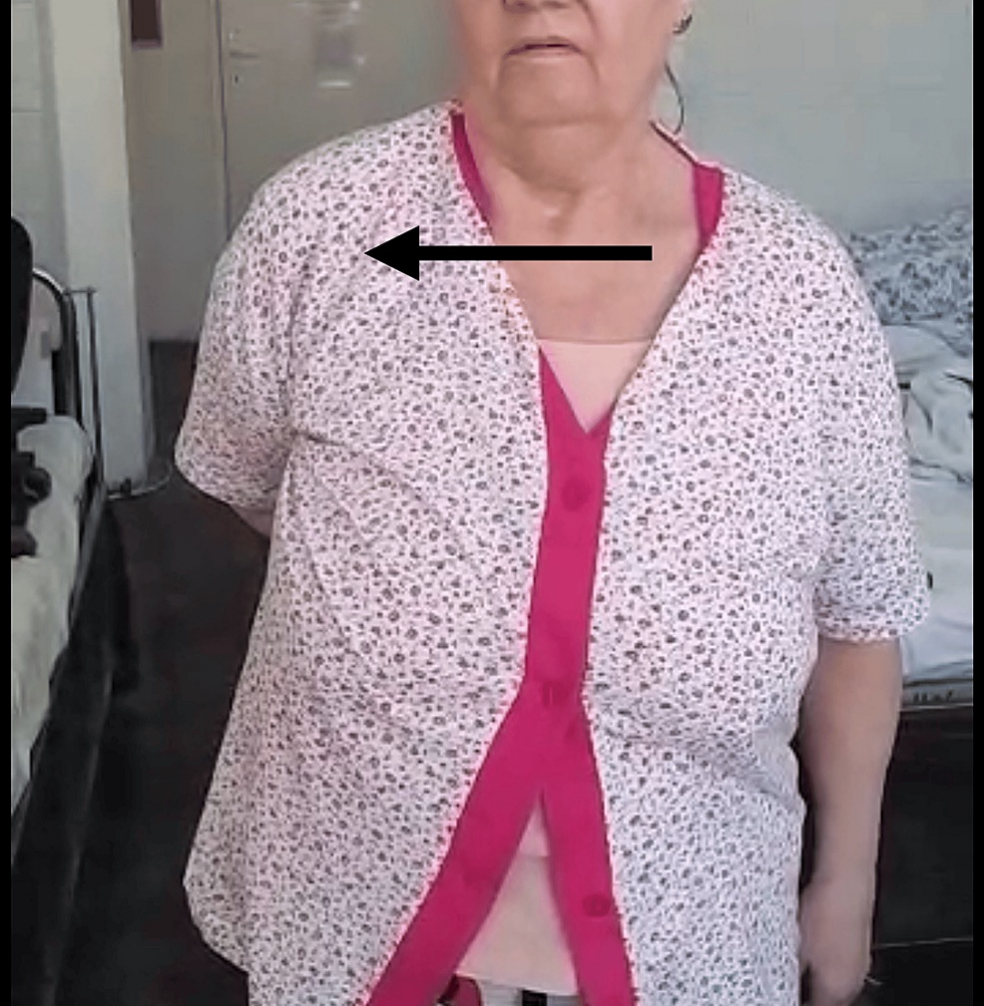
Patient range of motion at two years postoperatively The arrow shows the internal rotation of the right arm.

**Figure 5:**
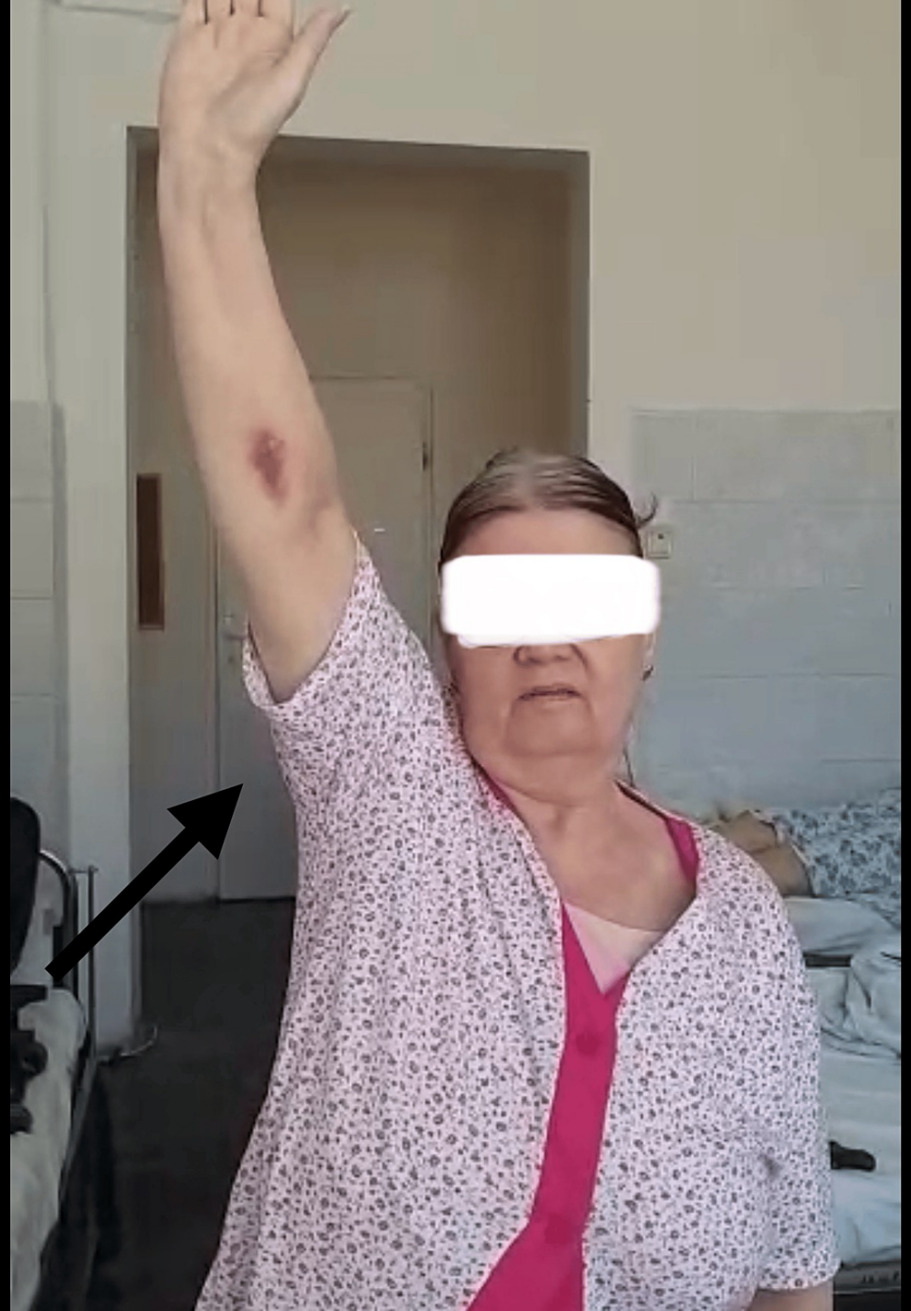
Patient range of motion at two years postoperatively The arrow shows the abduction of the right arm.

**Figure 6:**
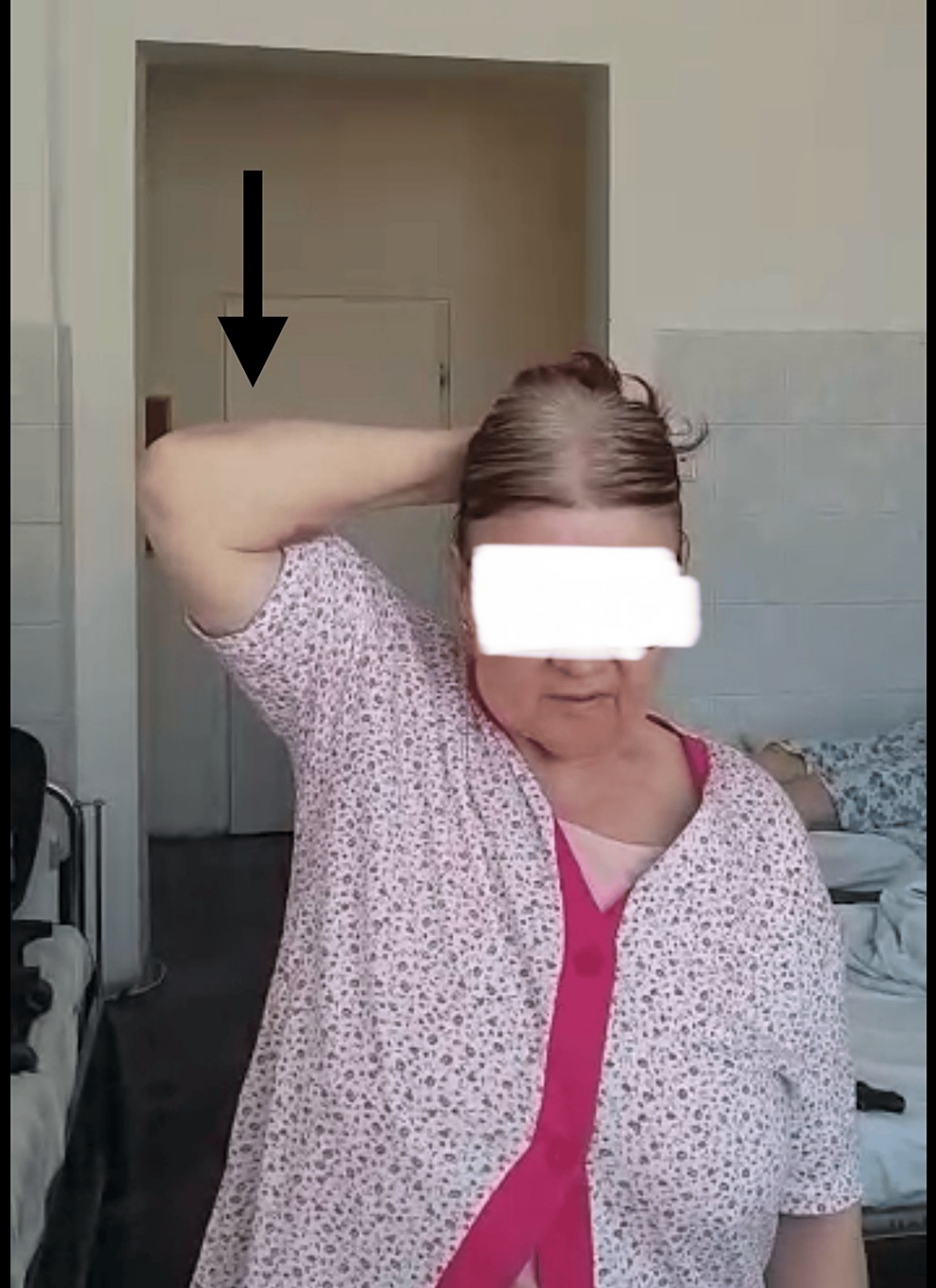
Patient range of motion at two years postoperatively The arrow shows the external rotation of the right arm.

At a postoperative follow-up at two years, the patient presented with a full, painless range of motion (Figures 4-6). The patient had no pain while sleeping and could carry out normal daily activities just as before the accident. The patient could carry a weight of 4-5 kg with the operated arm without pain, could manage toileting and get dressed, and could take care of herself with ease. Concerning the functional outcome scales, the results are as follows: (1) Neer’s “limited goals”: the patient reported no pain in the daytime as well as at night, was pleased with the result of the intervention, and was capable of taking care of herself; (2) ASES final score of 98.3: the patient declaring no pain and ease of daily activities; and (3) Constant-Murley score of 91: slight deficiency in internal rotation, can internally rotate up to waist-T12 (Figure 4), and slight deficiency in power. This result is a great leap from an ASES final score of 18.3 before surgery, when the patient had extreme pain, almost no range of motion, pain at night and daytime, and could carry out normal daily activities with great difficulty, relying on the contralateral upper limb. At two weeks postoperatively, the patient reported moderate pain and could execute a 30°-40° of arm abduction with the help of the contralateral hand. At six weeks, the patient had no pain, and arm abduction was almost 90° with moderate help. At six months, there was no pain and only a slight deficit in abduction and external rotation. 

## Discussion

Proximal humerus fractures are a common affliction in the elderly population and can be treated by various means. They can be treated either nonoperatively or conservatively if they fit these criteria: the fracture is a NEER two or three nondisplaced both in older and young patients; nondisplaced NEER two, three, or four in the elderly population; a slightly displaced fracture where the risks of anesthesia are too high [4]; minimal involvement of the articular surface [5]; and the patient is unfit for surgery.

On the other hand, the indications for surgical intervention are as follows: the humeral head is dislocated; the head, although in the glenoid socket, is facing another way, either superiorly or posteriorly; the head is split; the greater tuberosity is above the humeral head or is posterior; high medial pillar instability shown by great varus angulation of the head; the fragments shift positions in repeated X-rays when treated conservatively; and slight head-shaft angulation or minimal displacement of greater tuberosity in young and active patients [4].

**Figure 7:**
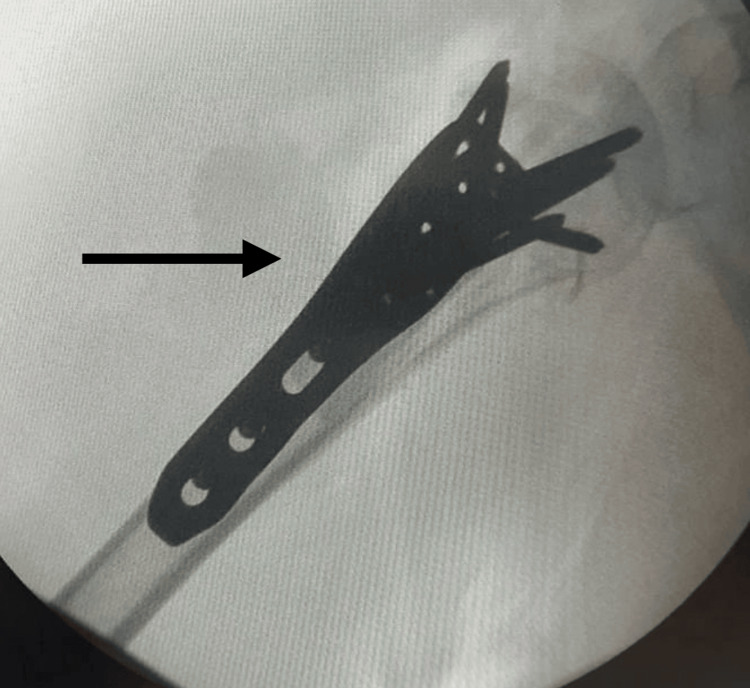
AP X-ray of the right shoulder revealing the right proximal humerus postoperatively The arrow shows the postoperative results: fracture reduction, plate, graft, and screw placement.

**Figure 8:**
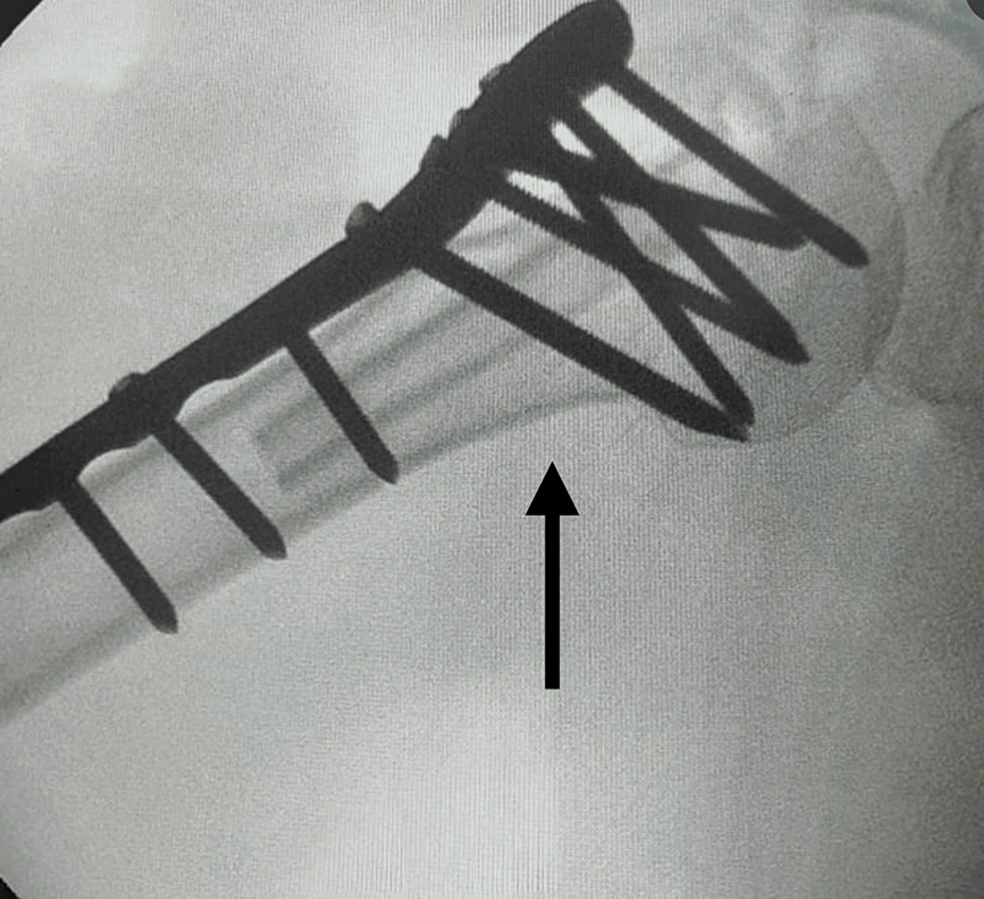
X-ray AP view of the right proximal humerus postoperatively The arrow shows postoperative results: fracture reduction, plate, graft, and screw placement with visible 6.5 cm of fibular autograft in the humerus shaft canal.

When considering operative treatment, one could take into account the following options: although percutaneous pinning is minimally invasive, its primary contraindications are head-splitting fractures, fractures of the anatomic neck [5], displacement, angulations, and most importantly, medial cortex comminution. Primary indications for percutaneous pinning are as follows: two-part fractures involving the surgical neck, lesser tuberosity, or greater tuberosity; three-part fractures involving the surgical neck and either the lesser or greater tuberosity; and impacted, valgus angulated four-part fractures [5]. Locking plates and screws is the treatment of choice for displaced NEER three or four fractures and fractures that do not meet the criteria for percutaneous pinning [5]. One of the drawbacks of locking plates and screws is a failure in case of a weak medial pillar given by medial calcar comminution [4]. Contrary to this, we can see a durable construct in our patient with a fibular autograft (Figures 7, 8). Arthroplasty is primarily taken into consideration in cases of head-splitting fractures that cannot be fixed, fractures with great valgus impaction, the comminution of the medial pillar, and fractures that lost all soft tissue attachments [5]. An advantage imposed by total reverse shoulder arthroplasty is that its performance does not depend on the healing of the tuberosities [5]. Contraindications for reverse shoulder arthroplasty are as follows: deltoid insufficiency, axillary nerve damage, and acromial or scapular associated pathology [5]. Indications for ORIF with locking plate and fibular autograft are medial calcar comminution and risk of varus malalignment. The main contraindication for fibular autograft is infection at the graft recipient site [6]. The majority of complications are generally concerning the donor site [6]. No absolute contraindications for fibula graft were found in the literature.

**Figure 9:**
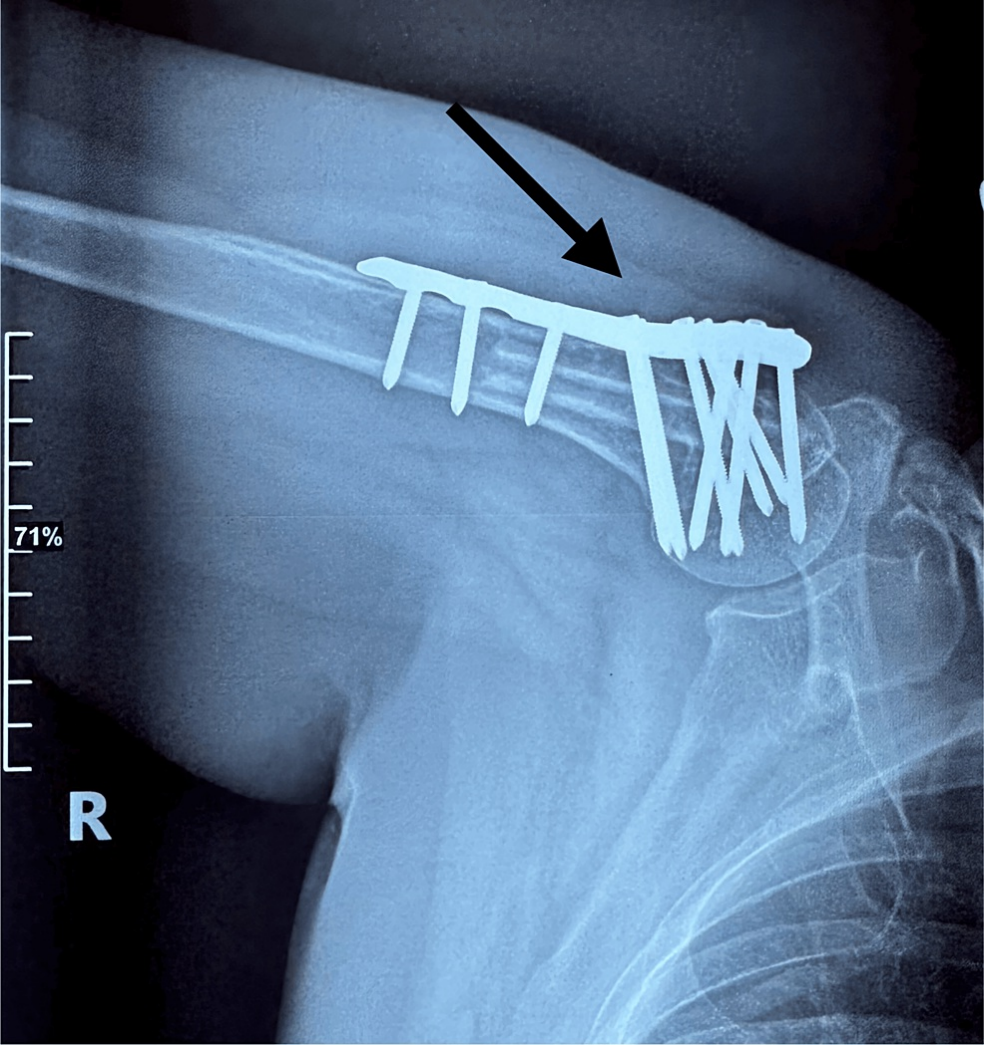
AP X-ray of the right proximal humerus at one and a half years postoperatively The arrow shows the right proximal humerus osteosynthesis construct in good condition.

**Figure 10:**
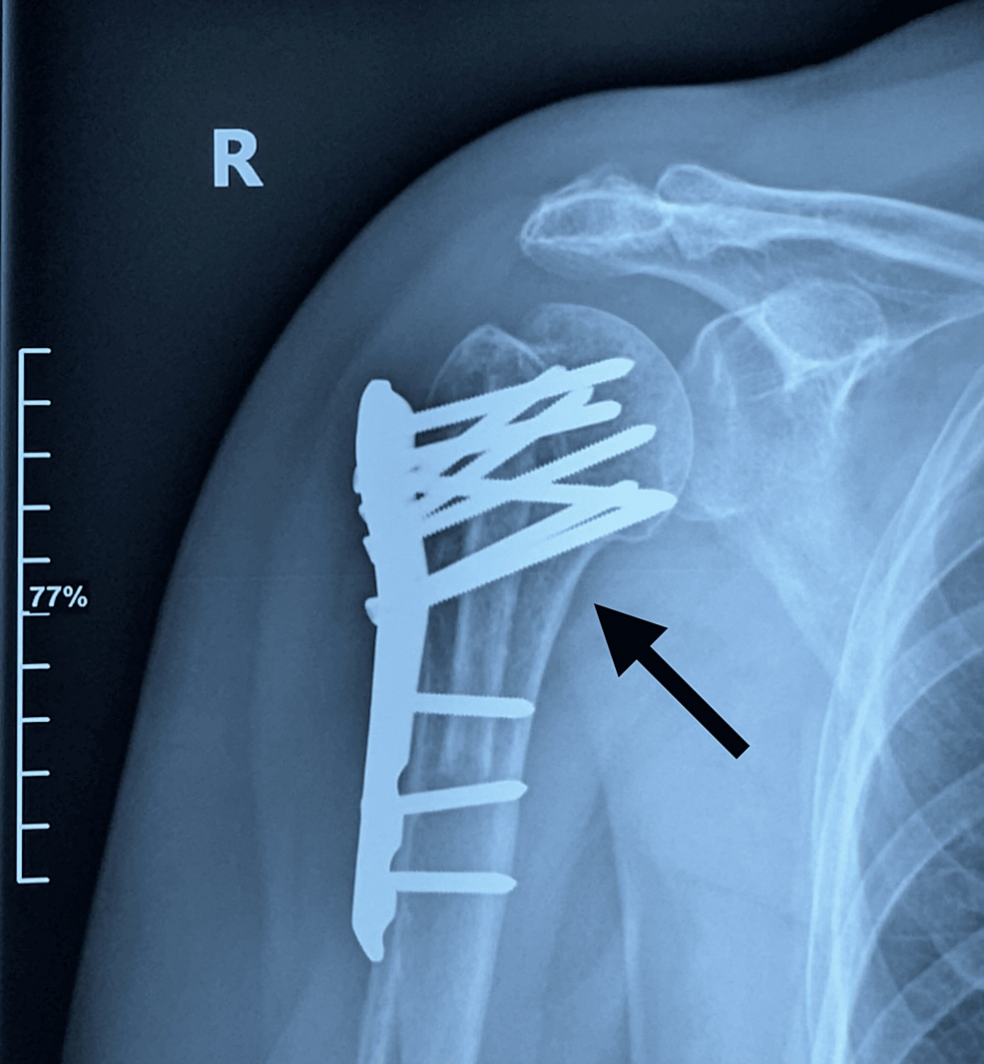
AP X-ray of the right shoulder at one and a half years postoperatively The arrow shows the right proximal humerus osteosynthesis construct in good condition.

**Figure 11:**
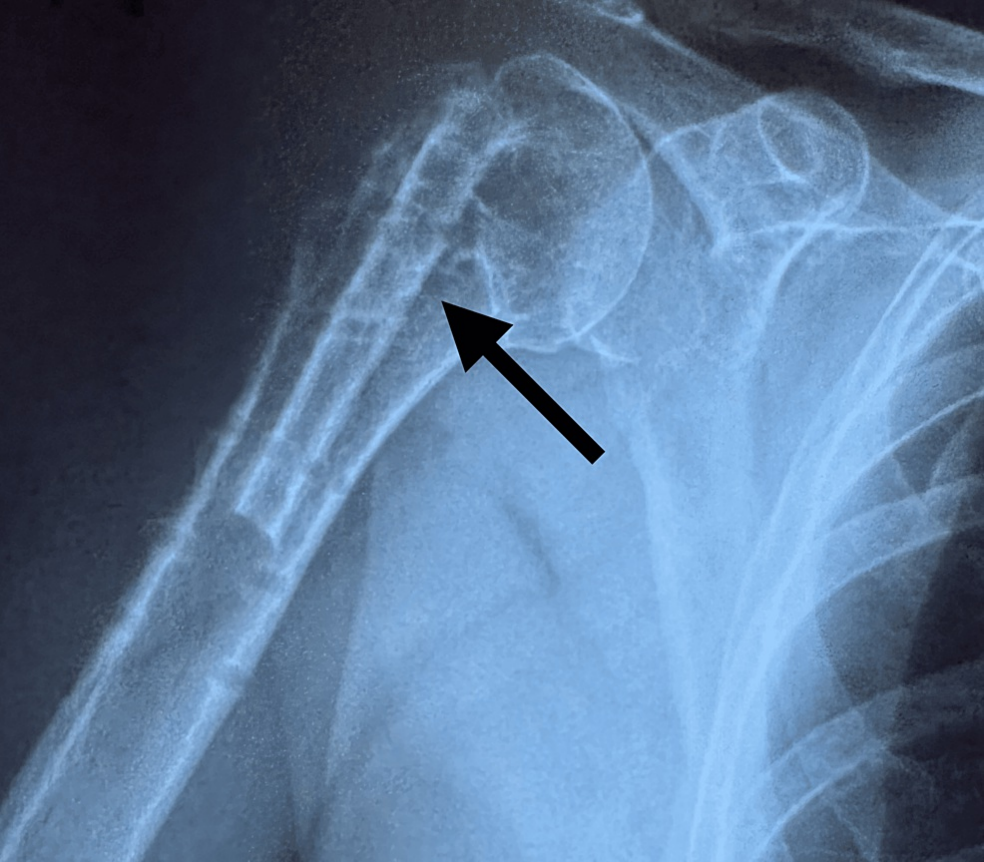
Postoperative X-ray of the right proximal humerus after surgical extraction of plate and screws at one and a half years from the first surgical intervention The arrow shows the right proximal humerus with the fibular graft and without the plate and screws.

In the case of our patient, we chose to opt for ORIF with locking plate and screws and fibular strut autograft due to it being the case of a patient under the age of 70, generally active and in good physical condition prior to injury with no history of preexisting shoulder ailment or pathologies implicating the rotator cuff or omarthrosis, which on the contrary would suggest the need of arthroplasty [5]. At one and a half years postoperatively, we can see our patient’s right proximal humerus osteosynthesis in good condition (Figures 9, 10) and also after the extraction of the plate and screws (Figure 11).

The medial wall comminution is a general factor of predisposition toward implant construct failure in the case of the sole use of a locking plate with screws [1]; therefore, we chose to use a fibular graft.

Our patient presented with no complications postoperatively and could start easy, passive pendulation movements the day after surgery. She had the plate extracted one and a half years after the operation.

## Conclusions

Our patient was an active 63-year-old woman with a NEER four fracture with severe loss of medial calcar. By keeping up with this particular patient with frequent follow-ups as well as a two-year observation period, we could see that our choice of an ORIF procedure with locking plate and screws and a fibular strut autograft was a viable method to prevent medial collapse and varus malalignment. This procedure was a good alternative to early reverse total shoulder arthroplasty in the case of this patient.

At a two-year follow-up, the patient presented with a painless full range of motion, was pain-free in the daytime and at night, and could carry out day-to-day activities pain-free.
